# Predictive value of albumin for intravenous immunoglobulin resistance in a large cohort of Kawasaki disease patients

**DOI:** 10.1186/s13052-023-01482-z

**Published:** 2023-06-25

**Authors:** Rui Zhang, Shuping Shuai, Hongxi Zhang, Jianghui Cai, Na Cui, Mi Tang, Shasha Xing, Yu Gao, Xia Liu, Xiao Yang

**Affiliations:** 1 Department of Pharmacy, School of Medicine, Chengdu Women’s and Children’s Central Hospital, University of Electronic Science and Technology of China, Chengdu, 611731 China; 2 Department of Emergency, Chengdu Women’s and Children’s Central Hospital, School of Medicine, University of Electronic Science and Technology of China, Chengdu, 611731 China; 3 School of Medicine, University of Electronic Science and Technology of China, Chengdu, 611731 China; 4 Office of Good Clinical Practice, School of Medicine, Chengdu Women’s and Children’s Central Hospital, University of Electronic Science and Technology of China, Chengdu, 611731 China; 5 Department of Obstetrics, School of Medicine, Chengdu Women’s and Children’s Central Hospital, University of Electronic Science and Technology of China, No.1314 Riyue Avenue, Qingyang District, Chengdu, 611731 China

**Keywords:** Kawasaki disease, Intravenous immunoglobulin, IVIG resistant, Albumin, Predictive value

## Abstract

**Background:**

Intravenous immunoglobulin (IVIG) has been the mainstay of treatment for Kawasaki disease (KD) over the past decades. However, 10–20% of KD patients are resistant to IVIG treatment which puts those patients at high risk of coronary artery lesions (CALs). Therefore, it is important to predict whether patients will be resistant to IVIG before the treatment. This study aimed to investigate the risk factors for IVIG non-responsive patients with KD.

**Methods:**

This study enrolled patients diagnosed with KD and divided them into two groups, IVIG responders and IVIG non-responders. We compared the differences in demographics and clinical data between the two groups. Differences among the groups were analyzed by ANOVA and Chi-square analysis. Predictors of IVIG resistance were determined by multiple logistic regression analysis and receiver operating characteristic (ROC) curve analysis.

**Results:**

In total, 907 KD patients were reviewed, with 841 IVIG responders and 66 IVIG non-responders. Patients in IVIG responders were younger than IVIG non-responders. The length of hospitalization of the IVIG non-responders was significantly longer than IVIG responders. The neutrophils%, C-reaction protein (CRP), and CRP/albumin ratio in IVIG responders were significantly lower than in IVIG non-responders (P < 0.05). The lymphocyte% and Albumin in IVIG responders were significantly higher than in IVIG non-responders. Multivariable logistic regression analysis demonstrated that albumin (OR = 0.881, 95% CI, 0.781 to 0.994, *p*-value = 0.039) was an independent risk factor for predicting IVIG resistance. The area under the ROC curve was 0.644, with a cut-off of ≤ 33.4 g/L determined by Youden’s index. The sensitivity and specificity in predicting IVIG resistance were 40.91% and 83.47%, respectively.

**Conclusion:**

Albumin can serve as a potential predicting marker for IVIG resistance in KD. A lower albumin level may be useful for identifying KD patients with a high risk of IVIG resistance to guide further therapy strategies.

**Supplementary Information:**

The online version contains supplementary material available at 10.1186/s13052-023-01482-z.

## Background

Kawasaki disease (KD) is an acute self-limiting inflammatory disorder associated with vasculitis, affecting predominantly medium-sized arteries, particularly the coronary arteries [1]. KD occurs primarily in children < 5 years of age and is now recognized as the most common cause of acquired heart disease in children in developed countries [2]. Intravenous immunoglobulin (IVIG) and aspirin have been established as the first-line therapy for KD to control inflammation and reduce the risk of coronary artery lesions (CALs) [3]. However, approximately 10–20% of KD patients who do not respond to the initial IVIG therapy are termed IVIG resistant [4]. IVIG resistance puts patients with KD at a higher risk for CALs and increases the treatment burden. Therefore, early prediction of initial IVIG resistance is vital in KD as those patients might benefit from an early-intensified therapy.

The systematic inflammatory response plays a critical role in the progression of KD, although the etiology of KD remains unknown [5]. Albumin (Alb), traditionally regarded as a marker of nutritional status [6], is a commonly used negative acute phase reactant. The albumin level is correlated with the severity of the acute inflammation, and hypoalbuminemia was commonly observed in KD patients during the acute phase [7]. Moreover, the C-reactive protein (CRP) to albumin (CRP/Alb, CAR) ratio has been considered a novel predicting marker for CALs formation and IVIG resistance in KD [8]. Thus, in this study, we aim to evaluate the predictive value of CPR, albumin, and CRP/ALB ratio for IVIG resistance in KD patients.

## Methods

The Chengdu Women’s and Children’s Central Hospital Ethics Committee approved the study protocol (Approval No. B202213) and waived informed consent requirements. All methods were carried out following the Declaration of Helsinki.

### Study design, setting, and study subjects

This was a retrospective cohort study. We retrospectively reviewed the clinical records of patients with KD hospitalized at Chengdu Women’s and Children’s Central Hospital from January 2018 to December 2020. All the KD patients (including complete and incomplete) were diagnosed according to the 2017 American Heart Association (AHA) guideline [1] and were confirmed by two experienced pediatricians. The inclusion criteria for study subjects were as follows: (1) patients were <18 years old; (2) patients of initial onset of KD; (3) patients received standard treatment with 2 g/kg of IVIG of single infusion within 10 days from fever onset. Exclusion criteria were as follows: (1) recurrent KD; (2) patients who had a serious infection, immune diseases, metabolic disease, hematological disease, or cardiovascular system diseases; (3) patients who had received IVIG within 6 months in other medical facilities; (4) patients who did not receive IVIG or initial IVIG dose was < 2 g/kg; (5) patients with malnutrition and nutritional imbalance; (6) incomplete clinical or laboratory information.

Complete KD was diagnosed in children with a fever and had four or more of the following major symptoms: (I) polymorphous exanthema, (II) erythema and cracking of lips, strawberry tongue, and/or erythema of oral and pharyngeal mucosa, (III) changes of the peripheral extremities, (IV) bilateral conjunctival congestion, and (V) unilateral cervical lymphadenopathy. Incomplete KD was defined as a child with a fever with < 4 major symptoms and compatible laboratory or echocardiographic findings [1].

### Treatment regimen

According to our institutional treatment protocol, all KD patients were treated with oral aspirin (30-50 mg/kg/d) and IVIG (2 g/kg) within 10 days from fever onset. The dose of aspirin was decreased to 3–5 mg/kg/day after defervesce for 48 h. The second IVIG dose (2 g/kg) was administered if patients had an initial IVIG resistance. For patients with repeated IVIG resistance, intravenous methylprednisolone (30 mg/kg/dose) was given for 3 consecutive days. No patients received additional therapy such as plasma exchange, infliximab, or cytotoxic agents.

Initial IVIG resistance was defined as those with persistent fever for at least 36 h after completion of initial IVIG infusion [1]. Repeated IVIG resistance was defined as recurrent or persistent fever for at least 36 h after the second IVIG treatment. The patients who met the inclusion criteria were classified into two groups according to whether they were responsive or non-responsive to initial IVIG: IVIG responders and IVIG non-responders.

### Data collection

Clinical and laboratory data were collected through medical record review. Clinical data such as age, sex, and time of the first dose of IVIG were collected. Laboratory data on admission pre-IVIG treatment was collected, including CRP, hemoglobin (Hb), white blood count (WBC), platelet count (PLT), percentage of neutrophils (N%), lymphocytes, serum alanine aminotransferase (ALT), serum aspartate aminotransaminase (AST), albumin. All blood samples were collected before the initial IVIG infusion. The CRP to albumin ratio (CAR) was calculated by dividing CRP by albumin collected before the initial IVIG treatment. We would use the highest level of CRP and the lowest level of albumin for the analysis of CAR if there were more than one result of CRP or albumin before the initial IVIG infusion.

### Statistical analyses

The normality of distribution of variables was checked using the Kolmogorov-Smirnov test. Continuous variables were expressed as mean ± standard deviations or median and IQR (25th, 75th percentile) if non-normally distributed. Categorical variables were expressed by presenting the frequency and proportion in each category. The Chi-square or Fisher’s exact test was applied to compare categorical variables. Student’s t-test or Mann–Whitney U-test was used for continuous variables. We performed multivariate logistic regression analysis to identify the independent predictors of IVIG resistance. The receiver operating characteristic curve (ROC) was analyzed to assess the predictive accuracy of independent predictors for IVIG resistance. We also performed subgroup analysis by dividing the KD patients into complete and incomplete KD. Data were analyzed using IBM SPSS Statistics 25.0 (SPSS, Chicago, IL, USA). For all analyses, P < 0.05 was considered to be statistically significant.

## Results

### Study selection

**Fig. 1:**
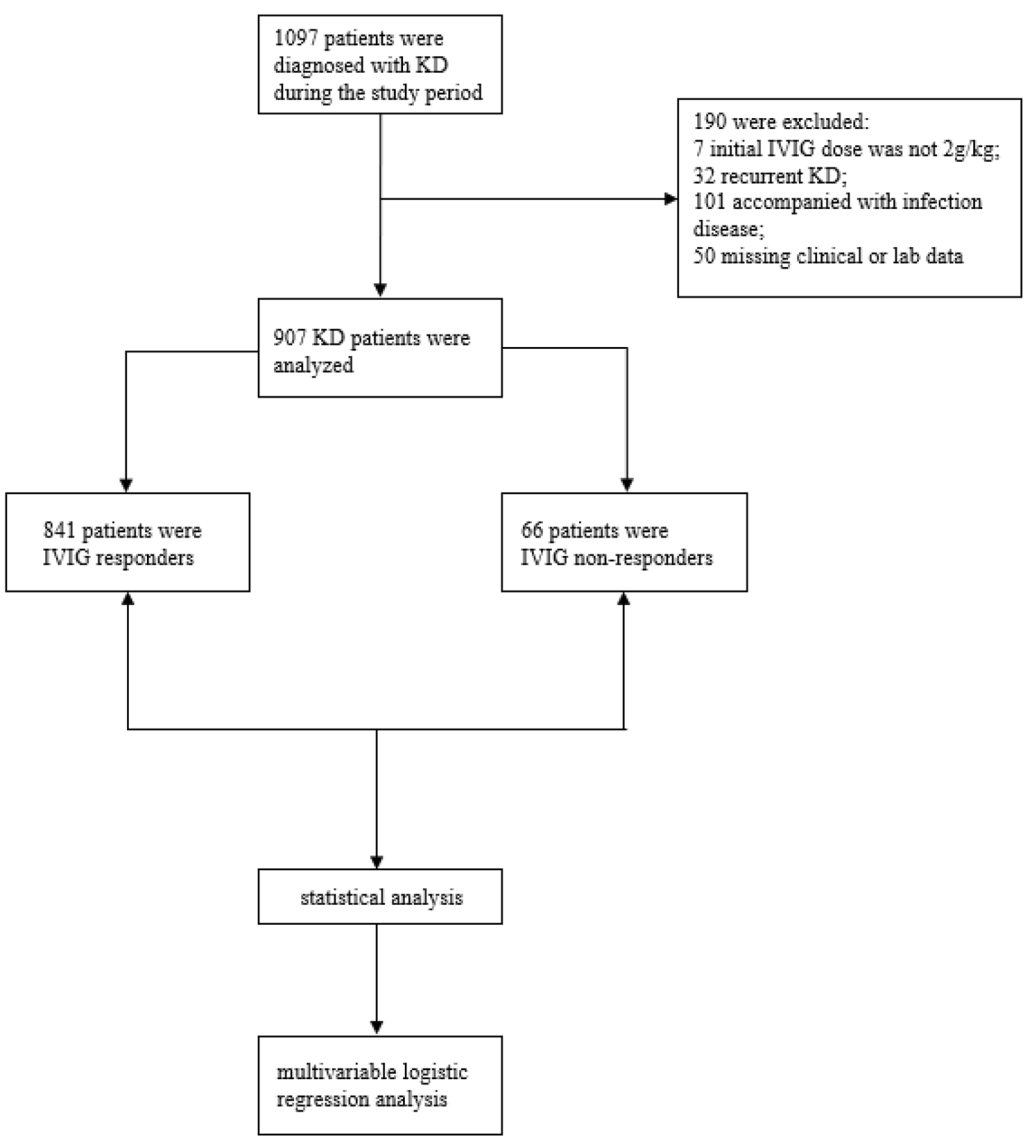
Flowchart of included KD patients

A total of 1097 children were diagnosed with KD during the study period. Seven received initial IVIG dose < 2 g/kg, 32 were recurrent KD, 101 had severe infection diseases, and 50 had incomplete clinical or laboratory data. After the exclusion, 907 KD patients who met the inclusion criteria were enrolled in this study (Fig. 1), including 841 IVIG responders and 66 IVIG non-responders.

## Characteristics of KD Patient

Table 1 shows the basic demographics and laboratory characteristics of the IVIG responders and non-responders groups. There were no significant differences in gender, diagnosis, WBC, HB, ALT, and AST. A slight difference (P = 0.042) was observed in PLT between the two groups. In addition, IVIG non-responders group had a longer length of hospitalization and older age. Compared with the IVIG responders group, the N%, CRP, and CRP/Alb were significantly higher in patients with IVIG resistance. On the contrary, lymph (%) and albumin were significantly lower in patients with IVIG resistance (Supplementary file, Appendix S1).

**Table 1:** Baseline characteristics of all KD patients

	IVIG responders (N = 841)	IVIG non-responders (N = 66)	*U* or *χ*^*2*^	*P*
**Age (month)**, Median (IQR)	23(12,38)	32.5(20,55)	-3.922	<0.001
**Gender**, n (%)				
male	497(59.1)	38(57.58)	0.058	0.897
female	344(40.9)	28(42.42)		
**Diagnose**, n (%)				
Complete KD	780(92.75)	62(93.94)	0.131	0.812
Incomplete KD	61(7.25)	4(6.06)		
**Length of hospitalization (day)**, Median (IQR)	7(6,8)	8(7,9)	-5.435	<0.001
**WBC (×10**^**9**^**/L)**, Median (IQR)	13.74 (10.75,17.13)	13.94 (10.47,16.5)	-0.462	0.644
**HB (g/L)**, Median (IQR)	108(101,115)	107.5(101,114)	-0.291	0.771
**PLT (×10**^**9**^**/L)**, Median (IQR)	356(286,446.5)	320.5(250,435)	-2.033	0.042
** N (%)**, Median (IQR)	64.7 (55.3,74.8)	72.8(58.4,83.5)	-3.246	0.001
L**(%)** Median (IQR)	25.2 (17.1,33.9)	18(11,29.3)	-3.613	<0.001
**CRP (mg/L)**, Median (IQR)	72(38,114.5)	105(66,151)	-4.236	<0.001
**ALT (IU/L)**, Median (IQR)	27.1 (15.05,68.25)	44.05(19,126.4)	-1.897	0.058
**AST (IU/L)**, Median (IQR)	32.2 (25.35,49.25)	36(25,63.8)	-1.02	0.308
**Albumin**	37.7 (34.9,40.2)	35.75(31,38.7)	-3.897	<0.001
**CRP/Alb**	1.914 (0.997,3.195)	3.099 (1.843,3.910)	-4.107	<0.001

KD = Kawasaki disease, IVIG = Intravenous immunoglobulin, WBC = White blood cell, HB = Hemoglobin, PLT = platelet, N%=percentage of neutrophils, L%=percentage of lymphocytes, CRP = C-reactive protein, ALT = alanine aminotransferase, AST = aspartate aminotransferase, Alb = Albumin.

### Characteristics of complete and incomplete KD patients

There were 842 complete KD patients, with a median age of 23 (13, 38.5) in IVIG responders and 32.5 (20, 55) months in IVIG non-responders. Patients in IVIG responders were younger than IVIG non-responders (P<0.01). The ratio of males to females was 1.44:1. Among all the complete KD patients, 780 were classified as IVIG responders, and 62 were IVIG non-responders. The length of hospitalization of the IVIG non-responders was significantly longer than IVIG responders. The N%, CRP, and CRP/Alb ratio in IVIG responders were significantly lower than in IVIG non-responders (P < 0.05). The lymphocyte% and albumin in IVIG responders were significantly higher than in IVIG non-responders. There were no statistical differences in PLT, WBC, HB, ALT, and AST among the two groups. Detailed information on the characteristics of complete KD patients can be seen in the Supplementary file, Appendix S2. There were 65 incomplete KD patients, with 61 IVIG responders and 4 IVIG non-responders. There were no statistical differences between the IVIG responders and IVIG non-responders in the incomplete KD, except the HB (Supplementary file, Appendix S3).

### Multivariate logistic analysis and ROC curve for predicting IVIG-resistance

**Fig. 2:**
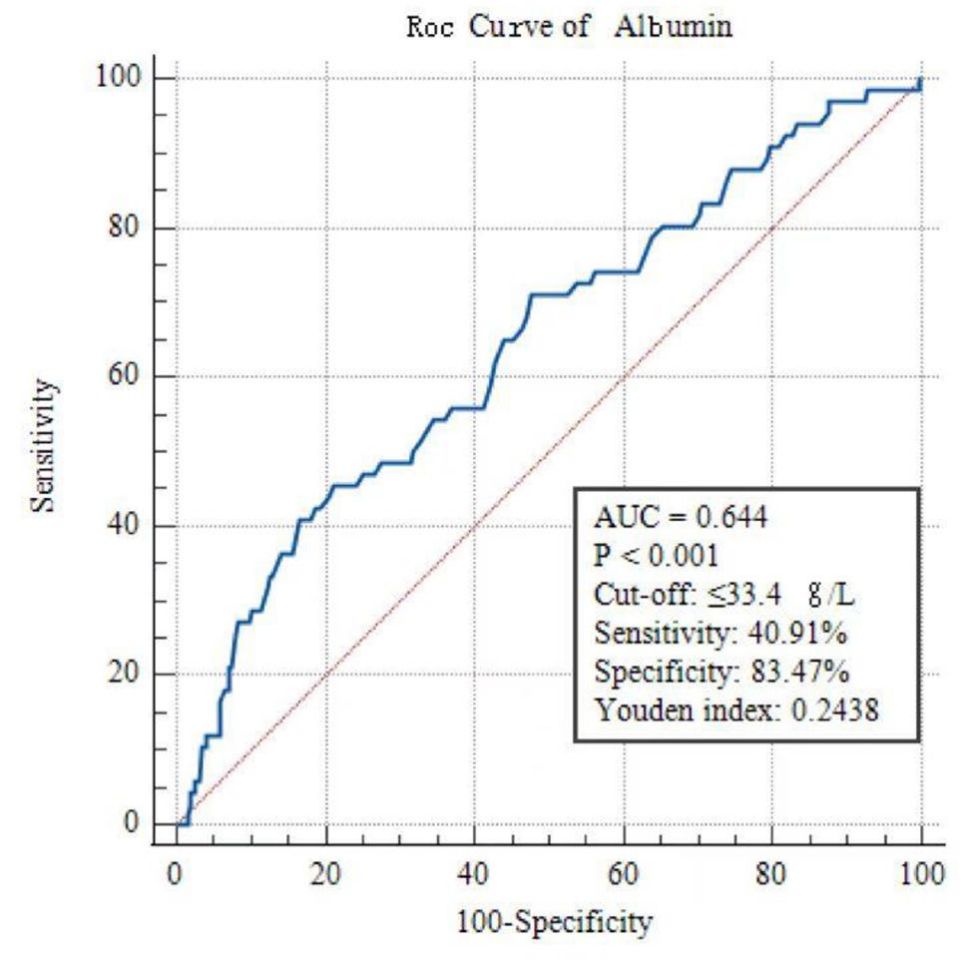
The ROC curve analysis of albumin for the prediction of IVIG-resistance.

Statistically significant variables were enrolled in the multivariate logistic regression from the univariate analysis. It was identified that lower albumin was an independent risk factor for IVIG resistance (Table 2). The area under the ROC curve (AUC) was 0.644, with a cut-off of ≤ 33.4 g/L determined by Youden’s index. The sensitivity and specificity in predicting IVIG-resistance were 40.91% and 83.47%, respectively (Fig. 2). The multivariate logistic analysis in complete KD was shown in the Supplementary file, Appendix S4. No significant differences were found using multivariable logistic regression analysis among the laboratory parameters that had revealed statistical differences in complete KD.

**Table 2:** Multivariable logistic regression analysis for predicting IVIG-resistance in all KD patients

	B	SE	Wald	P Value	OR	95% CI	
						lower	upper
Age (month), Median (IQR)	0.02	0.007	7.984	0.005	1.021	1.006	1.035
Length of hospitalization (day), Median (IQR)	0.44	0.088	24.752	0	1.552	1.305	1.846
PLT (×109/L), Median (IQR)	0	0.001	0.003	0.954	1	0.998	1.002
N (%), Median (IQR)	-0.007	0.014	0.234	0.629	0.993	0.966	1.021
L (%), Median (IQR)	-0.012	0.018	0.394	0.53	0.989	0.954	1.025
CRP (mg/L), Median (IQR)	0.014	0.018	0.616	0.433	1.014	0.979	1.05
Albumin	-0.127	0.062	4.258	0.039	0.881	0.781	0.994
CRP/ALB	-0.263	0.615	0.183	0.669	0.769	0.23	2.567

PLT = platelet, CRP = C-reactive protein, N%=percentage of neutrophils, L%=percentage of lymphocytes, TC = Total cholesterol, ALB = Albumin.

## Discussion

In the present study, the authors aimed to investigate the predictive value of laboratory parameters for initial IVIG resistance in KD patients. In the large retrospective cohort of 907 patients from southwestern China, univariable logistic regression analysis showed that N%, CRP, and CRP/Alb were significantly higher in the IVIG non-responders group than in the IVIG responders group. In addition, the lymph (%) and albumin were significantly lower in patients with IVIG resistance. Multivariable logistic regression analysis revealed that only lower albumin (≤ 33.4 g/L) before IVIG treatment was an independent risk factor (OR = 0.881, 95% CI, 0.781 to 0.994, *p*-value = 0.039) for initial IVIG resistance in KD patients. The AUC of albumin for predicting IVIG resistance in KD was 0.644, with a sensitivity and specificity value of 40.91% and 83.47%, respectively. Our study suggests that albumin is an independent predictor for IVIG resistance and may be useful in predicting initial IVIG resistance in KD patients.

Different clinical guidelines recommend IVIG as the first-line therapy to treat KD [1, 9]. Standard treatment with IVIG and aspirin significantly reduces the prevalence of CALs [10]. Nevertheless, about 10–20% of KD patients were IVIG-resistant and at a higher risk of developing cardiac complications [1]. Therefore, early prediction of IVIG resistance is one pivotal topic of interest and research hotspots in KD since those KD patients with IVIG resistance might improve an early-intensified therapy [11]. There are several Japanese (e.g., Harada score [12], Egami score [13], Kobayashi score [14]) or North American risk-scoring systems [15] for predicting IVIG resistance. However, these scoring systems are considered to be complicated and showed lower prediction effectiveness in non-Japanese populations [16–19]. Notably, the incidence of IVIG resistance in the present study was 7.28% (66/907), which was lower than the proportions reported in previous studies (10-20%) [20]. Accumulating evidence has found the incidence of IVIG resistance seems to be lower in China than in other countries [21–23].

The IVIG non-responders had a longer hospitalization and older age than the IVIG responders. The longer length of hospitalization may be due to the requirement of additional IVIG infusion or adjunctive therapies in IVIG non-responders. It is worth noting that patients in IVIG non-responders group are significantly older (mean age = 32.5 months) than IVIG-responders. Previous investigations revealed that ages under 1 year old [24, 25] or under 6 months [16] were risk factors for IVIG resistance. But a recent study [26] also reported older age (mean age = 27.6 months) was a risk factor for IVIG resistance. There are reasons to speculate that both older and younger age (e.g., ≤ 12 months) are risk factors for IVIG resistance. Furthermore, IVIG non-responders group had a higher level of N%, CRP, and CRP/Alb, which was consistent with previous studies [27–29]. The present data indicate more severe inflammation in KD patients with IVIG resistance during the acute phase. On the contrary, lymph (%) and albumin were significantly lower in patients with IVIG resistance.

Albumin was an independent predictor for IVIG resistance in this study. The present results were in line with previous studies, which reported similar findings [16, 22, 30]. Serum albumin, traditionally regarded as a maker of nutritional status, is also increasingly considered the most critical negative acute-phase protein [6]. Catabolism of albumin is directly correlated with the severity of acute inflammation [7]. Vascular leakage was supposed to be a key feature of KD pathophysiology, leading to hypoalbuminemia [7]. The vascular endothelial growth factor (VEGF) level can reflect the severity of vascular leakage. Elevation of Serum VEGF concentration was correlated with low serum albumin in KD patients [31].

However, albumin may not be suitable as a single predictor to accurately predict initial IVIG resistance in a clinical setting because of its low sensitivities (40.91%). The possible explanation is that albumin is related to the general inflammatory response but insufficient in reflecting an overall perspective of KD. Other factors, such as genetic, immune and IVIG metabolic, have not been considered. The genetic factor probably plays the basis of the differences in the individual responses to IVIG therapy. However, subgroup analysis showed albumin (OR = 1.012, 95% CI, 0.971 to 1.05, *p*-value = 0.513) was not an independent risk factor of IVIG resistance in complete KD. Thus, the results of subgroup analysis also partly explained that albumin might not be sufficiently reliable as a single indicator in predicting IVIG resistance. Meanwhile, a single inflammatory parameter may be easily influenced by other factors. Thus, a prediction model combined with various parameters may theoretically be more reliable and have the potential to be a powerful candidate marker to evaluate inflammatory status. Still, albumin is a cost-effective alternative that may provide additional information for IVIG resistance prediction in KD patients considering it is routinely measured in clinical practice as part of the complete blood count.

### Limitations

The present study was a retrospective study performed in a single hospital, which means potential selection or information bias may exist. Second, all participants were Chinese, which limits the generalizability of the results. Hence, further multicenter prospective studies are needed to verify the present results.

## Conclusions

KD patients with IVIG resistance had a significantly lower level of albumin. Low albumin is identified as an independent risk factor of IVIG resistance in KD patients. Further multicenter prospective studies are required to confirm the present results.

## Electronic supplementary material

Below is the link to the electronic supplementary material.

**Supplementary Material, Appendix S1**. Comparison of N%, L%, CRP, albumin and CRP/Alb between IVIG responders group and IVIG non-responders group. **Appendix S2**. Baseline characteristics of the complete KD patients. **Appendix S3**. Baseline characteristics of the incomplete KD patients. **Appendix S4**. Multivariable logistic regression analysis for predicting IVIG-resistance in complete KD patients

## Data Availability

The data used and/or analyzed during the current study are available within the manuscript.

## Abbreviations

IVIG: Intravenous immunoglobulin
KD: Kawasaki disease
CALs: Coronary artery lesions
ROC: Receiver operating characteristic curve
CRP: C-reaction protein
PLT: Platelet
WBC: White blood cell
Hb: Hemoglobin
Alb: Albumin
ALT: Serum alanine aminotransferase
AST: Serum aspartate aminotransaminase
AUC: Area under curve
AHA: American Heart Association
VEGF: Vascular endothelial growth factor
