# Supplementary material for: Predictive value of albumin for intravenous immunoglobulin resistance in a large cohort of Kawasaki disease patients
Source: Ital J Pediatr. 2023 Jun 25;49:78. doi: 10.1186/s13052-023-01482-z (PMC10291809; doi:10.1186/s13052-023-01482-z)
Supplement: Supplementary file 1 — Supplementary Material, Appendix S1. Comparison of N%, L%, CRP, albumin and CRP/Alb between IVIG responders group and IVIG non-responders group. Appendix S2. Baseline characteristics of the complete KD patients. Appendix S3. Baseline characteristics of the incomplete KD patients. Appendix S4. Multivariable logistic regression analysis for predicting IVIG-resistance in complete KD patients [file 13052_2023_1482_MOESM1_ESM.docx]

**Contents of supplementary file :**

1. **Comparison of N%, L%, CRP, albumin and CRP/Alb between IVIG responders group and IVIG non-responders group.**
2. **Baseline characteristics of the complete KD patients.**
3. **Baseline characteristics of the incomplete KD patients.**
4. **Multivariable logistic regression analysis for predicting IVIG-resistance in complete KD patients.**

**Appendix S1. Comparison of N%, L%, CRP, albumin and CRP/Alb between IVIG responders group and IVIG non-responders group.**


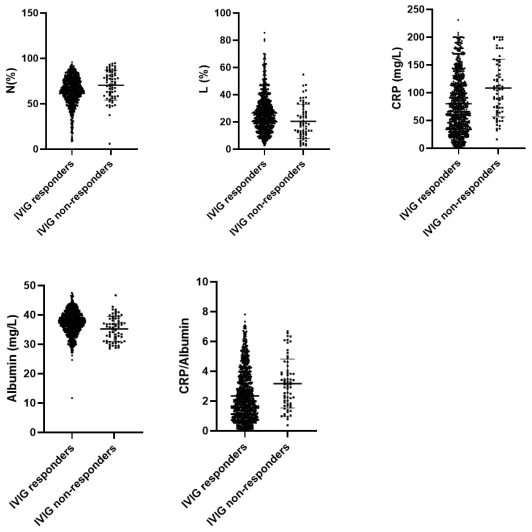


**Appendix S2. Baseline characteristics of the complete KD patients.**

|  | **IVIG responders**  **(N=780)** | **IVIG non-responders**  **(N=62)** | ***U* or *χ^2^*** | ***P*** |
| --- | --- | --- | --- | --- |
| **Age (month)**, Median (IQR) | 23(13,38.5) | 32.5(20,55) | -3.887 | 0 |
| **Gender**, n (%) |  |  |  |  |
| male | 460(58.97) | 37(59.68) | 0.012 | 0.914 |
| female | 320(41.03) | 25(40.32) |  |  |
| **Length of hospitalization (day)**, Median (IQR) | 7(6,8) | 8(7,9) | -5.34 | 0 |
| **WBC (×10^9^/L)**, Median (IQR) | 13.76  (10.81,17.165) | 13.83  (10.25,16.28) | -0.926 | 0.355 |
| **HB (g/L)**, Median (IQR) | 109(102,115) | 108(103,115) | -0.13 | 0.897 |
| **PLT (×10^9^/L)**, Median (IQR) | 351(284,438) | 322.5(243,438) | -1.652 | 0.099 |
| **N (%)**, Median (IQR) | 65.6  (56.3,75.2) | 72.8(58.8,82.3) | -2.923 | 0.003 |
| **L(%)**  Median (IQR) | 24.4  (16.8,33.7) | 18(11.5,27.5) | -3.34 | 0.001 |
| **CRP (mg/L)**, Median (IQR) | 72(39,115) | 106(64,155) | -4.057 | 0 |
| **ALT (IU/L)**, Median (IQR) | 27.8(15,70.95) | 44.05  (16.7,105.1) | -1.571 | 0.116 |
| **AST (IU/L)**, Median (IQR) | 32.1  (25.4,49.45) | 35.75  (23.4,63.8) | -0.712 | 0.477 |
| **Albumin** | 37.7  (35.05,40.2) | 35.85  (31.2,38.7) | -3.756 | 0 |
| **CRP/Alb** | 1.924  (1.027,3.217) | 3.099  (1.806,4.049) | -3.902 | 0 |

KD=Kawasaki disease, IVIG=Intravenous immunoglobulin, WBC=White blood cell, HB=Hemoglobin, PLT=[platelet](javascript:;), N%=percentage of neutrophils, L%=percentage of lymphocytes, CRP=C-reactive protein, ALT = alanine aminotransferase, AST = aspartate aminotransferase, Alb=Albumin.

**Appendix S3. Baseline characteristics of the incomplete KD patients.**

|  | **IVIG responders**  **(N=61)** | **IVIG Non-responders**  **(N=4)** | ***U* or *χ^2^* or t** | ***P*** |
| --- | --- | --- | --- | --- |
| **Age (month)**, Median (IQR) | 14.5(7,36.5) | 26.5(5,58) | -0.314 | 0.753 |
| **Gender**, n (%) |  |  |  |  |
| **male** | 37(60.66) | 1(25) | 2.236 | 0.135 |
| **female** | 24(39.34) | 3(75) |  |  |
| **Length of hospitalization (day)**, Median (IQR) | 8(6,9) | 8.5(7.5,9) | -1.041 | 0.298 |
| **WBC (×10^9^/L)**, Median (IQR) | 13.52±4.6 | 17.22±3.41 | -1.577 | 0.12 |
| **HB (g/L)**, Median (IQR) | 104.2±12.06 | 91±11.69 | 2.124 | 0.038 |
| **PLT (×10^9^/L)**, Median (IQR) | 440.59±166.09 | 318±56.76 | 1.461 | 0.149 |
| **N (%)**,  Median (IQR) | 56.13±12.15 | 61.66±40.41 | -0.273 | 0.803 |
| **L (%)**  Median (IQR) | 29.25  (25.7,36.75) | 19.65  (5.4,38.95) | -0.792 | 0.429 |
| **CRP (mg/L)**, Median (IQR) | 61.5  (34.5,94.5) | 93.5(77.5,102) | -1.332 | 0.183 |
| **ALT (IU/L)**, Median (IQR) | 20.7(15.65,48) | 77.85(24.6,138) | -1.583 | 0.113 |
| **AST (IU/L)**, Median (IQR) | 32.5  (25.1,44.2) | 47.9(34.7,85.7) | -1.392 | 0.164 |
| **Albumin** | 36.19±4.29 | 33.23±5.09 | 1.328 | 0.189 |
| **CRP/Alb** | 2.08±1.51 | 2.76±0.75 | -0.886 | 0.379 |

KD=Kawasaki disease, IVIG=Intravenous immunoglobulin, WBC=White blood cell, HB=Hemoglobin, PLT=[platelet](javascript:;), N%=percentage of neutrophils, L%=percentage of lymphocytes, CRP=C-reactive protein, ALT = alanine aminotransferase, AST = aspartate aminotransferase, Alb=Albumin.

**Appendix S4. Multivariable logistic regression analysis for predicting IVIG-resistance in complete KD patients.**

|  | B | SE | Wald | P Value | OR | 95% CI | |
| --- | --- | --- | --- | --- | --- | --- | --- |
|  |  |  |  |  |  | Lower | Upper |
| **Age (month)**, Median (IQR) | 0.021 | 0.007 | 7.883 | 0.005 | 1.021 | 1.006 | 1.036 |
| **Length of hospitalization (day)**,  Median (IQR) | 0.454 | 0.091 | 25.003 | 0 | 1.575 | 1.318 | 1.882 |
| **PLT (×109/L)**, Median (IQR) | 0.001 | 0.001 | 0.412 | 0.521 | 1.001 | 0.999 | 1.003 |
| **N (%)**, Median (IQR) | -0.005 | 0.015 | 0.094 | 0.759 | 0.995 | 0.966 | 1.025 |
| **L(%)**, Median (IQR) | -0.007 | 0.019 | 0.16 | 0.689 | 0.993 | 0.957 | 1.029 |
| **CRP (mg/L)**, Median (IQR) | 0.012 | 0.019 | 0.428 | 0.513 | 1.012 | 0.976 | 1.05 |
| **Albumin** | -0.124 | 0.064 | 3.759 | 0.053 | 0.884 | 0.78 | 1.001 |
| **CRP/ALB** | -0.202 | 0.643 | 0.099 | 0.753 | 0.817 | 0.232 | 2.883 |

PLT=[platelet](javascript:;), CRP=C-reactive protein, N%=percentage of neutrophils, L%=percentage of lymphocytes, TC=Total cholesterol, ALB=Albumin.
